# Energy saving potential analysis applying factory scale energy audit **–** A case study of food production

**DOI:** 10.1016/j.heliyon.2023.e14216

**Published:** 2023-03-02

**Authors:** Derar Al Momani, Yousef Al Turk, Mohammed I. Abuashour, Haris M. Khalid, S.M. Muyeen, Tha’er O. Sweidan, Zafar Said, M. Hasanuzzaman

**Affiliations:** a Department of Allied Engineering Sciences, Faculty of Engineering, The Hashemite University, Zarqa, Jordan; b Alternative Energy Technology Department, Al-Zaytoonah University of Jordan, Queen Alia Airport St 594, Amman, Jordan; c Renewable Energy Centre, The Hashemite University, Zarqa, 13115, Jordan; d Department of Electrical and Electronics Engineering, Higher Colleges of Technology, Sharjah, 7947, United Arab Emirates; e Department of Electrical and Electronic Engineering Science, University of Johannesburg, Aukland Park 2006, South Africa; f Department of Electrical Engineering, University of Santiago, Avenida Libertador 3363, Santiago, RM, Chile; g Department of Electrical Engineering, Qatar University, Doha, 2713, Qatar; h Sustainable and Renewable Energy Engineering Department, College of Engineering, University of Sharjah, PO Box 27272, Sharjah, United Arab Emirates; i U.S.-Pakistan Center for Advanced Studies in Energy (USPCAS-E), National University of Sciences and Technology (NUST), Islamabad, Pakistan; j Department of Industrial and Mechanical Engineering, Lebanese American University (LAU), Byblos, Lebanon; k Higher Institution Center of Excellence (HICoE), UM Power Energy Dedicated Advanced Center (UMPEDAC), University of Malaya, Jalan Pantai Baharu, 59990, Kuala Lumpur, Malaysia

**Keywords:** energy Audit, Energy-efficiency, Boiler efficiency, Energy saving, Energy-efficient electric motors

## Abstract

An energy audit (EA) is a crucial step in boosting factory energy efficiency and obtaining certification for cleaner manufacturing. The results of a preliminary energy audit carried out at a sizable industrial facility in Jordan that creates some of the most well-known foods in the Middle East are presented in this study. The monthly demand of the factory for diesel ranged from 75,251.545 to 166,666.67 L. The factory energy model which is used to examine the impact of various energy-saving practices on the factory’s primary energy consumption, was developed with the help of the energy audit. It has been established that optimizing the factory’s energy use and the boiler systems' performance with regards to diesel consumption can withstand an expected monthly financial savings of 14205.85 Jordanian Dinar (JD). This has allowed a reduction in energy use of up to 18%. The CO_2_ harmful emissions were also decreased. Additionally, it is estimated that switching from the proposed motors to energy-efficient motors will cost less overall over time, saving around 3472.314 JD/month or 0.33576/year on average. Moreover, it was discovered that a total of 772.82021 Ton CO_2_/year emissions may be avoided each year.

## Highlights

• Industrial energy efficiency – Main element in economy changeover.
• Energy Audit (EA) – A requirement in decreasing barriers to energy efficiency.
• EA diagnoses the operating problems which affect energy efficient operations.
• An EA strategy devised on large-scale industry factory of food production.
• EA strategy features: 1) audit objectives, 2) scope, and 3) methodology at the food production plant.

## Graphical abstract

**Figure:**
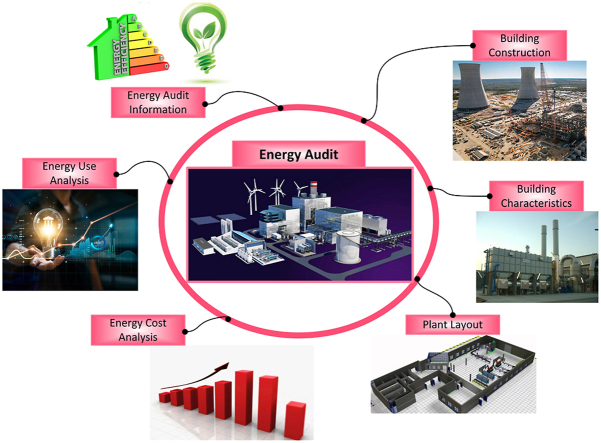

## 1 Introduction

The Jordanian national development policy now places a strong emphasis on energy saving and industrial pollution reduction. Due to its significance in the goal of low energy consumption, enhanced economic competitiveness, and the reduction of CO_2_ emissions, energy efficiency improvement has attracted increasing attention in many industrial sectors. Numerous research has been done from various angles to reduce energy use and CO_2_ emissions in the food industry. The depletion of fossil fuels has made the approach to energy consumption more cautious. The variants of renewable energy interaction in the form of vehicle-to-grid (V2G) systems [1,2], energy-water nexus [3], renewable energy integration (REI) [4,5], PV interaction [[6], [7], [8]] have been actively utilized to address the energy intermittency. In this pursuit, unjustified energy consumption in a high standard energy-efficient and sustainable environment is critical in the industrial sector. To resolve this issue, an EA is required which can generate an analysis of energy flows towards energy conservation in an industrial establishment referred mainly by the 1) higher inductive loads, 2) leakage, 3) isolation, 4) ventilation losses, and 5) environmental hazards resulting from pollution [[9], [10], [11], [12], [13]].

Industrial energy efficiency is the main element in the changeover of the economy concerning improved sustainability. For an industrial company, shrinking energy budgets is realized through applying energy-efficient tools, energy-saving revisions, load control, and more energy-efficient actions and measures. The elimination of existing market obstacles and inadequacies hinder the proficient end-use of energy audits. This requires a vital tool in decreasing barriers to energy efficiency in the form of EA to significantly implement the in-house energy controlling program in the industry [14], which is the motivation of this paper, moreover finding solutions to lower operational costs or the amount of energy consumed per unit of product output and thus achieving a potential saving is the main rationale for this energy audit in the factory.

**Fig. 1:**
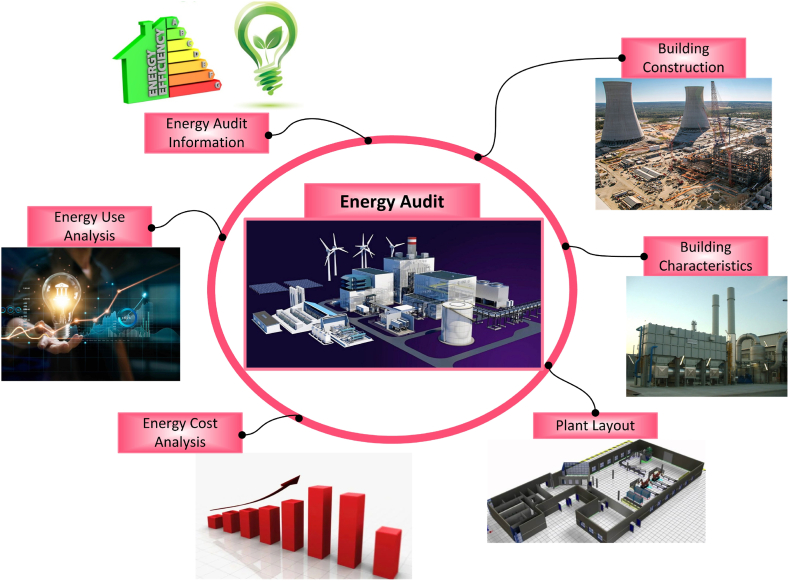
Graphical abstract of this article.

EA is an adequate practice to optimize energy in industrial sites and buildings while diagnosing the operating problems that could affect an energy-efficient operation [15,16]. In the past years, study about energy wastage saving has become a trend and evolved due to energy consumption that leads to a negative influence on the environment [17]. Energy-efficient designs and construction of buildings and properties of the controlled construction of energy decision-makers on the accomplishment of energy-efficiency for the construction sector is a vital and urgent role [[18], [19], [20]]. The modeling of industrial enterprise capacity audits based on energy and resource efficiency assessments and the development of a manufacturing model that produces resource-saving and energy-savings modeling, as well as clarification of the basic definitions of energy management information, is studied extensively [21]. Achieving best practices for energy saving in manufacturing areas is important for the awareness that energy saved is energy produced and that much economical cost and saving issues [22]. Exploring the results of energy-efficiency enhancements and investigating the profits attained under several energy-efficiency actions and best practices from energy audits based on case studies in different fields are investigated [23]. The consumption of energy profile of a standard wheel rim industrial plant and a set of maintenance, and economic issues. This may be applied in a complete analysis to achieve energy efficiency in manufacturing to support the selection of the best existing technology [24]. The analysis of the EA of food processing manufacturing and purification and bottling corporation in Ota, Nigeria was commenced to recognize the main causes of energy in use, find the breaks in energy usage, classify areas to advance energy usage, define the level of consumption of the energy sources, and endorsing policy actions to improve energy savings in the industries sector [25]. The main strategies for reducing energy use and greenhouse gas emissions in the Swedish timber sector. This relates to the examination of the technical possibilities for energy efficiency at the process level, as well as the classification of processes and the energy KPIs [26]. Eight sizable industrial buildings owned by a famous Italian automobile manufacturing group had a preliminary energy audit performed on them. The site’s buildings had heating energy demands that ranged from 6 to just over 74 kWh/m^3^/year. The factory energy model was created with the help of the energy audit in order to examine the effects of different energy-saving measures on the primary energy consumption of the location and the results were impressive [27]. Using dry process rotary kiln technology, the thermal energy audit analysis of a new generation pyro-processing unit for a cement factory in Iran was conducted. The findings identified the parts of the pyro-processing unit where thermal energy is lost and demonstrated that there is a good balance between the total input and output heat energy [28]. The EA data study of condiment manufacturing in India was explored. This study chiefly emphasizes the approximation of the load factor, energy use, energy savings, and yearly bill savings with a repayment period of the electric motors of the factory Moreover, there were several motors running under loaded circumstances notwithstanding the non-availability of variable frequency drives (VFDs) mounted in the factory [29]. The outcomes of an energy audit in meat processing manufacturing and the comprehensive and practical approaches to energy-saving actions to identify issues that can regulate a possible changeover to sustainable outlines of electricity consumption is addressed [30]. Consideration is consequently turned to the energy efficiency and energy audit issues defining the employment, approval, and spread of these enhancements [31,32]. Energy-efficiency improvements are used in this metaphorical sense to include any measure that results in the delivery of energy usage with a reduction of energy consumption [[31], [32], [33]]. The energy conservation in textile industries was studied in Ref. [34]. The policy mechanism of energy efficiency was discussed in Ref. [35]. The industrial boiler energy efficiency and its audit was discussed in Refs. [[32], [33], [34], [35], [36]]. The assessment criteria and energy efficiency of motors and their characteristics were discussed in Refs. [37,38]. The industry energy case studies and their performance measures and best practices were discussed in Refs. [[33], [34], [35], [36], [37], [38], [39]]. The focus of this paper is to devise EA for a large-scale industry factory of food production in Al Zarqa district of Jordan while enhancing their energy resources. A graphical abstract of this article can be seen in Fig. 1.

The main contribution of this work is to identify the: 1) audit objectives, 2) scope and 3) methodology at the Al Kasih Factories Group plant. The audit objective is to identify the main areas of energy-saving opportunities at the Al Kasih Factories Group plant. The scope of the energy audit was to visit the plant and take measures and energy data, then analyze this data and come up with steps to elevate the save opportunities. The energy audit methodology was a walk-through audit with energy surveys and analysis while looking to find the energy and cost-saving venues.

**Fig. 2:**
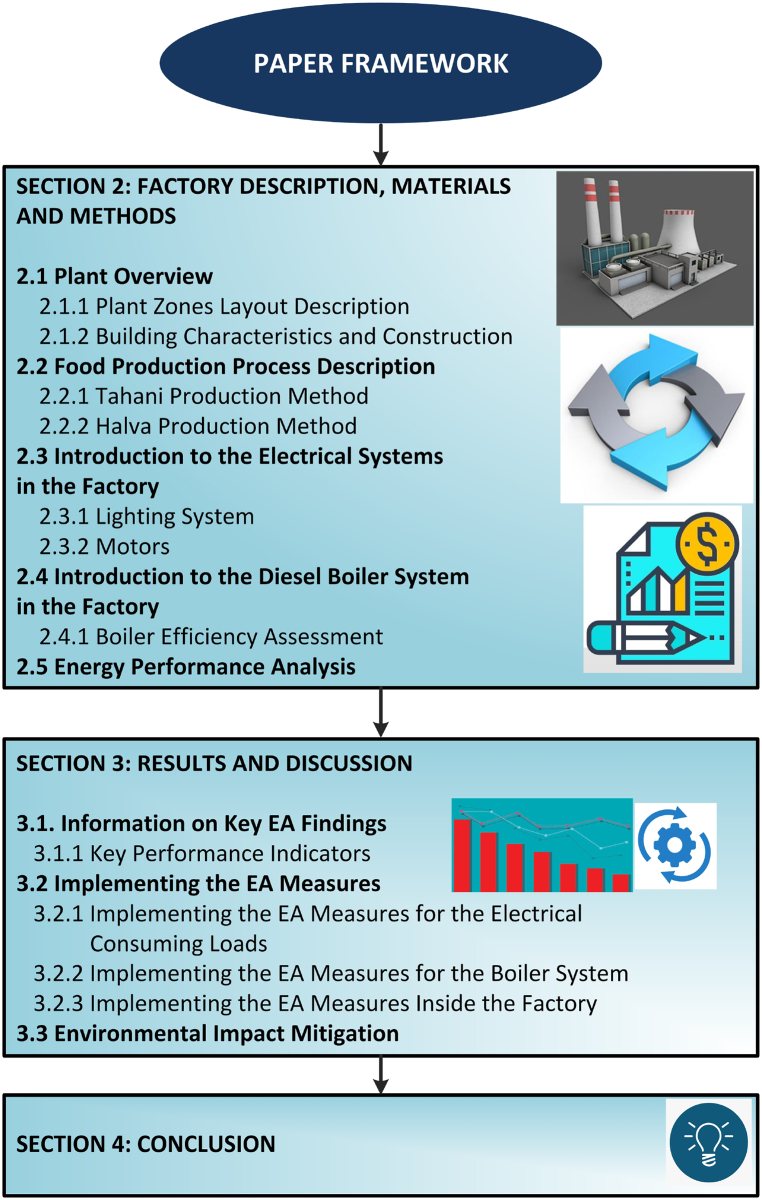
Framework of the paper.

The paper is structured as follows: Section 2 addresses the factory description, materials, and methods. This involves the machines utilized and the methods conducted on the machines. Section 3 presents the results and discussions on the implementation of the EA measures. The conclusion is drawn in Section 4. The framework of the paper can be seen in Fig. 2.

## 2 Factory description, materials, and methods

This section describes the factory from different sides such as 1) a general description of the factory, 2) the machines used, and 3) the methods conducted on these machines as a start-up to conduct the energy audit procedures while optimizing the energy usage for achieving energy efficiency and best practices.

### 2.1 Plant overview

**Fig. 3:**
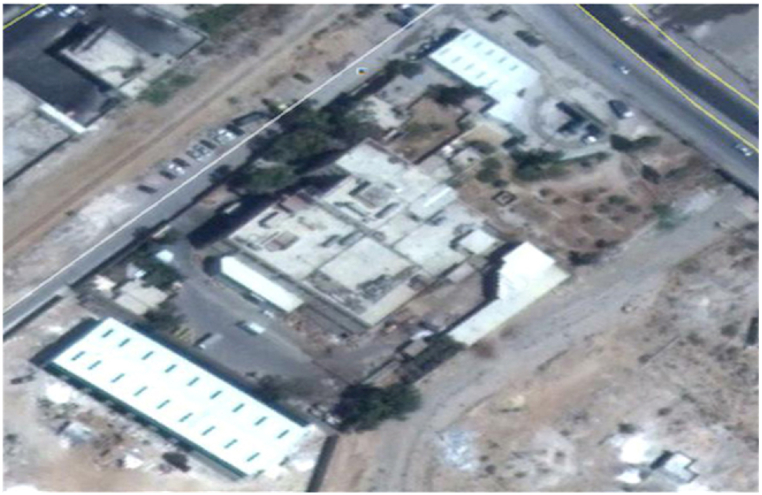
Plant top view – Coordinates (32.0608° N, 36.0942° E).

In this section, the plant under study is overviewed. The plant is in the Al-Zarqa district, Jordan. The top view is shown in Fig. 3. The coordinates of the plant are 32.0608° N, 36.0942° E*.* The plant was established in 1996 on a land area of 16,470 m^2^. The plant’s constructed area is 2390 m^2^. The plant is divided into 9 zones. It uses steam for the sesame drying and roasting. Moreover, a set of electric motors is utilized in the production of Tahini and Halva. Note the plant overview is for Al Kasih Factories Group. The Al Kasih Factories group confirms an informed consent for 1) making the factory information available, 2 process data available, and 3) possible publication of this article built on the information collected.

#### 2.1.1 Plant zones layout description

**Fig. 4:**
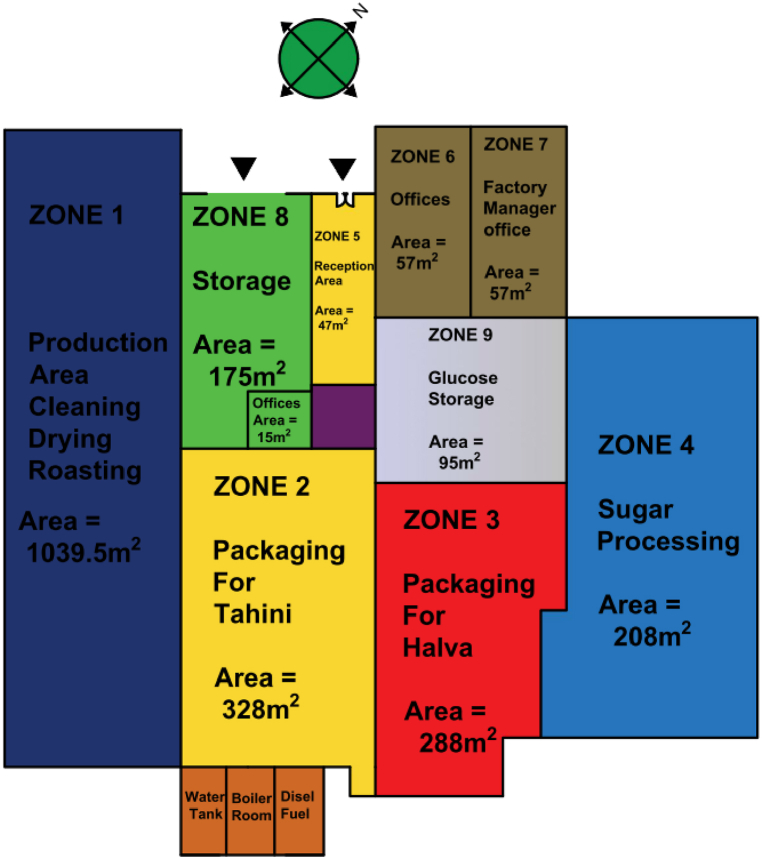
Plant zones layout.

**Fig. 5:**
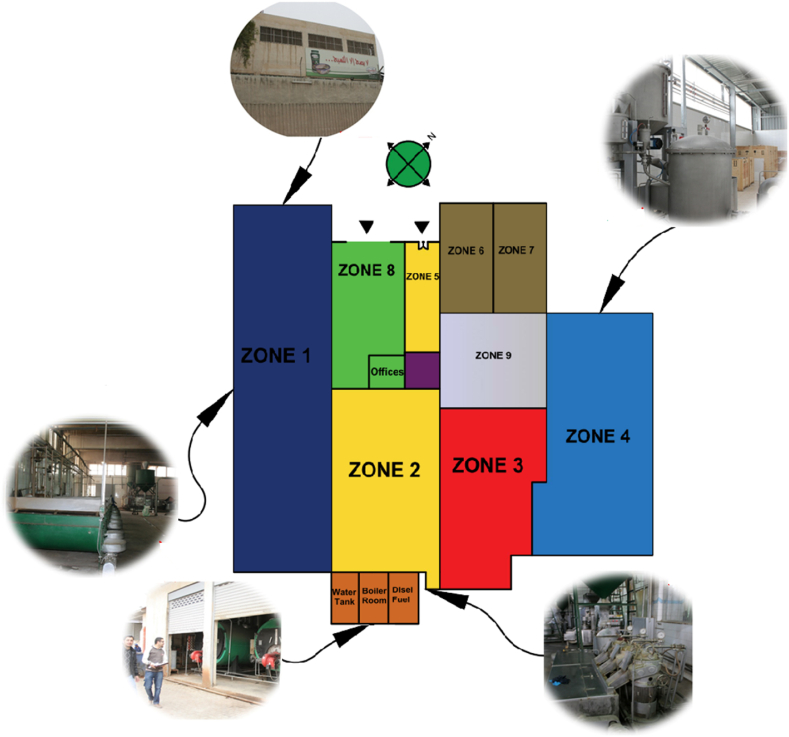
Plant different locations pictures.

The plant zone layout can be seen in Fig. 4, Fig. 5. The zone-wise description can be seen as follows.

1) *Zone 1:* This is the production area for cleaning, dehulling, roasting, and milling. The working hours of this zone are 24 h a day, and 7 days a week.
2) *Zone 2:* This is the parking area for the Tahini. Here the packing and labelling process also takes place. The working hours of this zone are 8 h a day, 6 days a week.
3) *Zone 3:* This is the parking area for Halva. Here the packing and labelling process takes place. This zone has working hours of 8 h a day, and 6 days a week.
4) *Zone 4:* Zone 4 is the sugar processing area where the process of cooking and dissolving the sugar as well as adding the Halva plant roots – “Saponaria officinalis” takes place. This zone has working hours of 8 h a day, and 6 days a week.
5) *Zone 5:* This is a reception area. It has work timings of 8 h a day, and 6 days a week.
6) *Zone 6:* This is the office area where the managerial work takes place. This zone has work timings of 8 h a day, and 6 days a week.
7) *Zone 7:* This is the office of the factory manager. It has working hours of 8 h a day, and 6 days a week.
8) *Zone 8:* This is the storage area where the finished products are stored for shipping. This zone has working hours of 8 h a day, and 6 days a week.
9) *Zone 9:* This is a sugar and glucose storage area. This zone has working hours of 8 h a day, and 6 days a week.
2.1.2 *Building Characteristics and Construction:* The main entrance of the building is oriented to the northwest which makes it away from direct sunlight in the summer. However, the production Zone area is on the south side which makes it exposed to direct sunlight for a more extended period. This can be analyzed by looking at Fig. 3, Fig. 4, Fig. 5 respectively.

The glazing is made on the four sides of the building which works as natural light for the plant during the day working hours. The windows are mostly fixed single-glazed with aluminium frames except for Zone 4, a steel structure extension to the old plant. The glazing for the Zone 4 area is made of fixed transparent fiberglass. There is no cooling system in the plant except for Zone 6 and Zone 7 which are the management offices. For Zone 6 and 7, a 2-ton split unit is utilized. The floors are made of cement except for Zone 5, 6, and 7 which are finished with ceramic. The walls are made of 20 cm prick with 2 cm plaster from inside and outside and no insulation between the bricks except the walls for Zone 4. The walls of Zone 4 are made of 0.5 mm pre-printed corrugated steel sheets with no insulation. The ceiling is constructed of 25 cm reinforced concrete. The windows are mostly fixed single-glazed windows with aluminium frames, which have been there since the opening of the plant in 1996. Therefore, they are not in good shape and require maintenance. The doors are made of steel. The main entrance door is made of steel. The storage door is made from a roll-up steel sheet with a thickness of 0.7 mm.

### 2.2 Food production process description

The method used to make tahini and halva is demonstrated in this section.

#### 2.2.1 Tahani production method

**Fig. 6:**
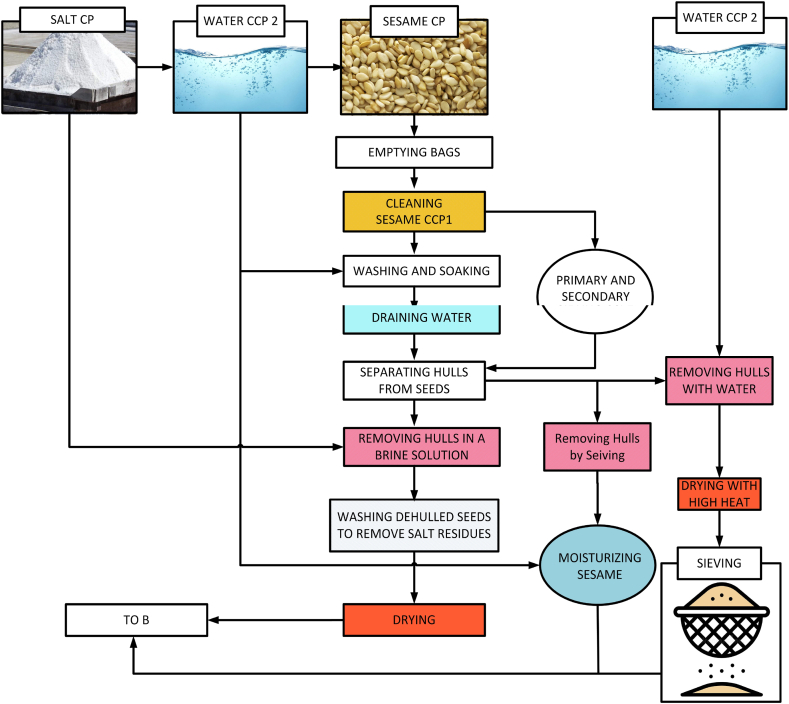
Sesame seed preparation and dehulling.

**Fig. 7:**
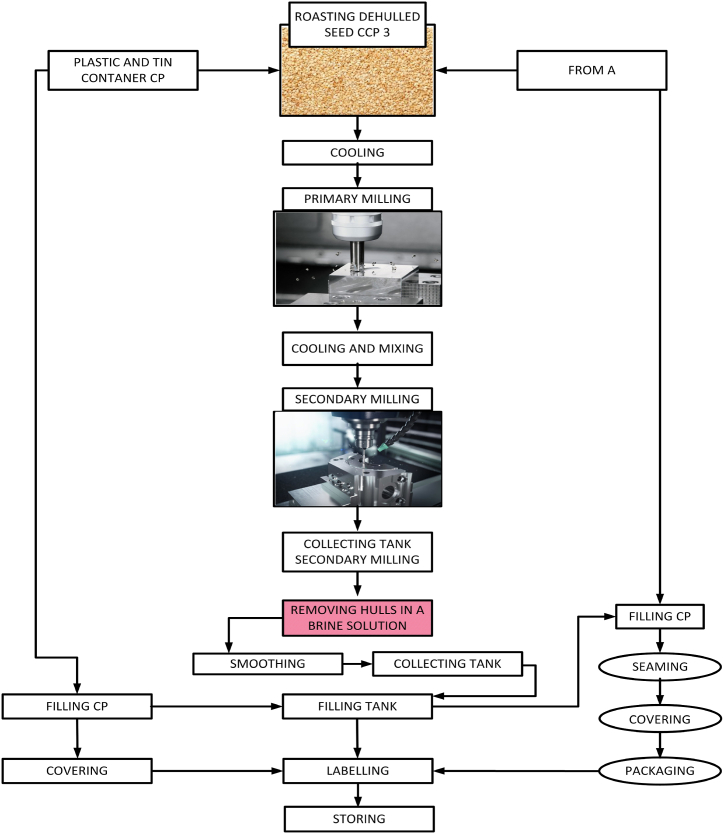
Tahini preparations.

The highest-quality sesame seeds are selected and stored in containers located in computer-programmed and controlled storerooms under special conditions. It is then selected and prepared for the Tahini production process. The sesame seed preparation and dehulling can be seen in Fig. 6. It is shifted through and cleaned of any dirt or dust particles so that it is as clean as possible and ready for production. This is followed by rinsing and peeling off the sesame. After being peeled, the sesame is roasted to elevate its taste giving the Tahini a longer shelf life. Then as depicted in Fig. 7, sesame undergoes another cleaning process for preparing Tahini and is grounded in special and large millstones while maintaining appropriate temperatures and high-quality production conditions. This delicate process is done mechanically through process critical control point (CCP) and control point (CP) without human interference. It is all carried out by sophisticated, precise, computer-programmed machines. After all stages of production are complete, very high-quality Tahini is made with innovative and precise machines. This is how a clean, clear, and high-quality product with high nutritional value is processed.

#### 2.2.2 Halva production method

**Fig. 8:**
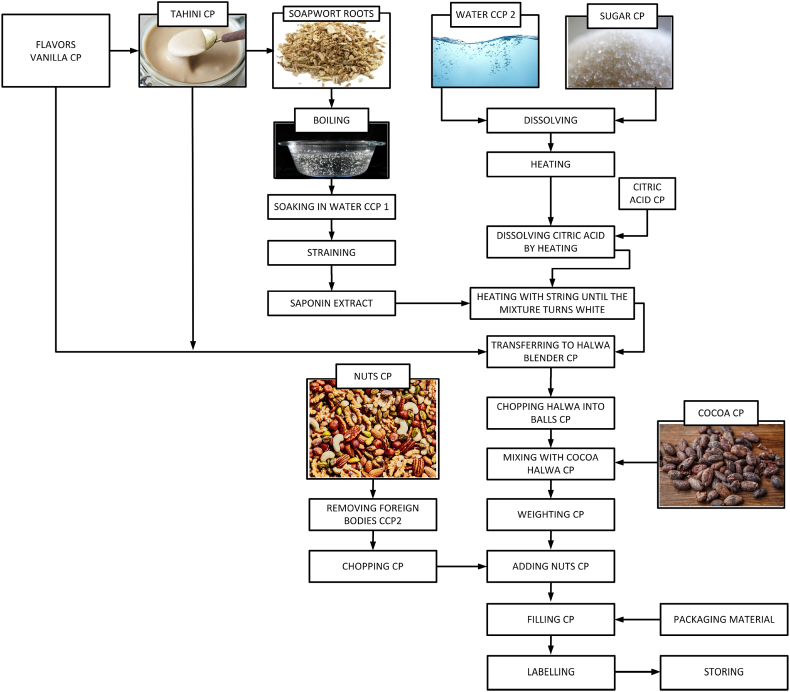
Halva preparations.

Halva preparations are made following the procedures shown in Fig. 8. It is processed from raw Tahini which is a mixture prepared through the cooking and dissolving process of various sweets as well as halva plant roots – “Saponaria officinalis” is further refined. Many different flavours can be added to this mixture such as: cocoa, pistachio, nuts, coffee, and more. All flavours are completely natural. The Halva is packaged in two sizes and weights: 1) 450 g, and 2) 900 g.

### 2.3 Introduction to the electrical systems in the factory

The electrical system is introduced in this section which involves the lighting system and motors.

#### 2.3.1 Lighting system

Projection lights and neon lighting are used for most of the plant and natural lighting in the morning hours for Zone 4. Note that the lux meter is used here for measuring brightness and intensity with which brightness appears to the human eye.

1) *Zone 1:* Zone 1 has a lux meter reading of 38 lux and the number of fixtures is 4 projection lights.
2) *Zone 2:* Zone 2 has a lux meter reading of 793 lux and the number of fixtures is 2 projections and 3 neon lights.
3) *Zone 3:* It has a lux meter reading of 450 lux. The number of fixtures is 4 projections and 2 neon lights.
4) *Zone 4:* Zone 4 has a lux meter reading of 995 lux. The number of fixtures is 2 projections and natural lighting.
5) *Zone 5:* Zone 5 has a lux meter reading of 455 lux. The number of fixtures is 2 neon lights.
6) *Zone 6:* Lux meter reading of 554 lux. The number of fixtures is 2 neon lights.
7) *Zone 7:* Lux meter reading of 650 lux. The number of fixtures is 2 neon lights.
8) *Zone 8:* Lux meter reading of 55 lux. The number of fixtures is 2 neon lights.
9) *Zone 9:* Lux meter reading of 32 lux. The number of fixtures is 1 neon light.

#### 2.3.2 Motors

**Fig. 9:**
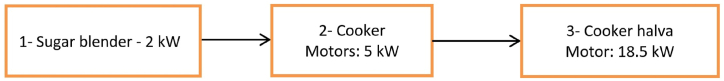
Motors in the department of cookers.

**Fig. 10:**
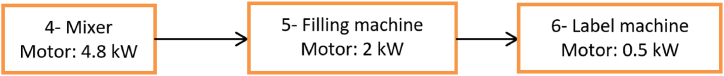
Motors in the department of mixers.

**Fig. 11:**
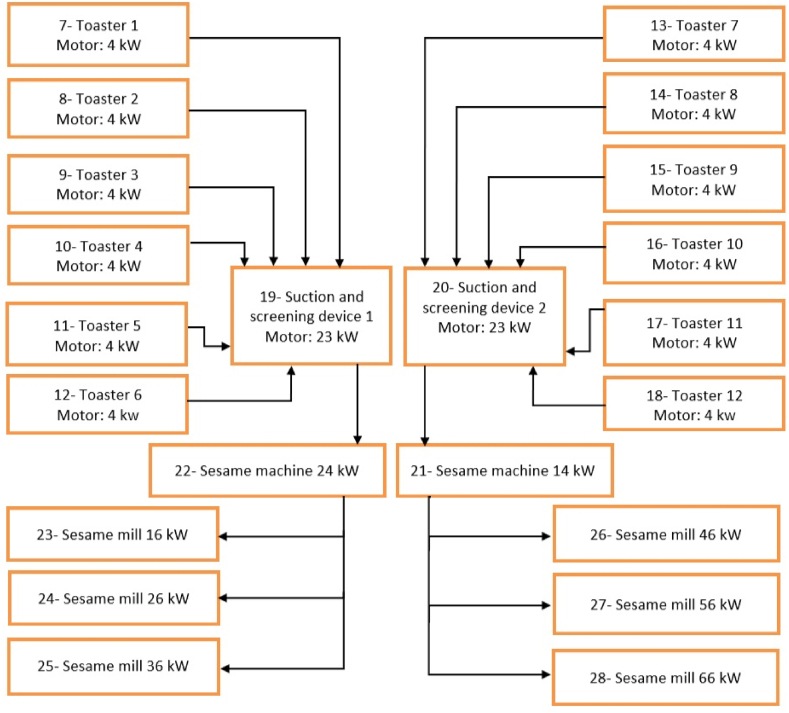
Motors in the department of roasting.

The 28 motors inside the factory are listed below in Table 1. This also includes details about the size, use, estimated hours of operation per year, and defining the motors that are forced into the rewinding process. This information is obtained through the maintenance team and recorded data inside the factory. The EA process and motor use will be explained in the block diagrams as shown in Fig. 9, Fig. 10, Fig. 11

1) Calculation of Annual Energy Saving: The annual energy saving (kWh) can be calculated knowing that the typical load factor (L.F) is 0.8 and the price in JD of kWh in the industrial sector is 0.081 JD using the following Eqs. (1), (2), (3) [35,40]:

Annual Energy Saving (kWh) = ((Motor Size × Working Hours/Year) × L.F)

(1) x*((*1/ER*)* *−* *(*1/EN*))* × *Motor Size*

where *ER* denotes the efficiency after the rewinding process percentage, and *EN* represents the new efficiency model percentage.

2) *Calculation of Annual Saving and Payback Period:* The annual saving and the simple payback period can be calculated as follows:

(2) *Annual saving (JD)* = *Annual energy saving kWh* × *0.081 JD*

(3) *Simple Payback Period (SPP) = Annual saving (JD)/Initial Cost*

**Table 1:** Motors information.

Motor No.	Size (kW)	Use	Motor Efficiency (%)	Motors Forced to the Rewinding Process	Working Hours/Year
1	2	Sugar Blender	91	_	2496
2	5	Cooker	91	_	2496
3	18.5	Cooker Halva	87	_	2496
4	4.8	Mixer	88	Rewinding	2496
5	2	Filling Machine	90	_	2496
6	0.5	Label Machine	90	_	2496
7	4	Toaster 1	90	_	8640
8	4	Toaster 2	90	Rewinding	8640
9	4	Toaster 3	90	_	8640
10	4	Toaster 4	90	_	8640
11	4	Toaster 5	90		8640
12	4	Toaster 6	90	Rewinding	8640
13	4	Toaster 7	90	Rewinding	8640
14	4	Toaster 8	90	Rewinding	8640
15	4	Toaster 9	90	_	8640
16	4	Toaster 10	90	_	8640
17	4	Toaster 11	90	_	8640
18	4	Toaster 12	90	_	8640
19	23	Suction and Screening Device 1	86	_	8640
20	23	Suction and Screening Device 2	86	Rewinding	8640
21	4	Sesame Machine 1	90	_	8640
22	4	Sesame Machine 2	90	Rewinding	8640
23	6	Sesame Mill 1	89	_	8640
24	6	Sesame Mill 2	89	Rewinding	8640
25	6	Sesame Mill 3	89	Rewinding	8640
26	6	Sesame Mill 4	89	_	8640
27	6	Sesame Mill 5	89	_	8640
28	6	Sesame Mill 6	89	Rewinding	8640

### 2.4 Introduction to the diesel boiler system in the factory

The boiler is the heart of the heating system and generates thermal energy by burning diesel fuel. In some months, the cost of buying oil is mounted to around 100,000 JD. The factory’s boiler generates steam, which is mostly used to roast sesame seeds to enhance their flavour and extend the shelf life of Tahini. The steam is circulated throughout the facility via pipes. The gathered data reveals that the heating system consumes about 90% of the total energy bill.

#### 2.4.1 Boiler efficiency assessment

The boiler efficiency has always been questioned due to the overheating impact on evaporation rate decrease over time, damage to the heat transfer system, and poor performance and maintenance. Even in new boilers, reasons such as deterioration of fuel quality, water quality, etc. Can lead to poor performance of the boiler. The assessment of boiler efficiency can be observed in (4)–(18).

1) Calculation of Boiler Efficiency: Boiler efficiency testing helps detect boiler efficiency deviations from maximum efficiency and target problem area to correct action. Boiler heat efficiency is defined as the percentage of heat input used to generate steam and is given in Eq. (4) as follows:

(4) *Boiler Efficiency* = *m*^*o*^*steam* × *Cp* × *(T*_*steam*_ − *T*_*fw*_*)*/*m*^*o*^*diesel x GCV*

where T_fw_ is the feedwater temperature, T_steam_ is the saturated steam temperature, Cp is the specific heat of superheated steam (0.45 kcal/kg °C). m^o^ diesel is the diesel consumption rate. m^o^ steam is the steam consumption rate and (GCV) is the gross caloric value per kcal/kg of fuel. There are reference standards for boiler testing on-site namely British standard (BS) 845: 1987, and USA standard, American Society for Mechanical Engineers (ASME) Power Test Code (PTC)-4-1 generating units' [23].

**Fig. 12:**
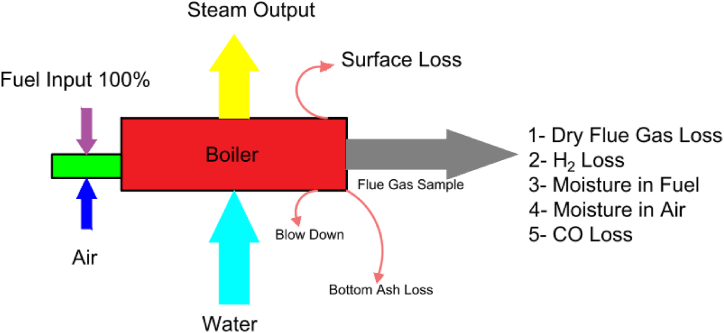
Losses that occur in a boiler.
4) *Heat Losses Percentage Equation:* The percentage heat loss due to dry flue gas is expressed in Eq. (5) as:

2) *Principles of Boiler Loss:* The principles of boiler loss are: 1) loss of heat due to dry gas, 2) loss of heat due to moisture in hot air, 3) loss of heat due to hydrogen burning, 4) loss of heat due to radiation, 5) loss of heat due to non-combustion, 6) loss due to moisture in the fuel, and 7) losses due to hydrogen combustion depends on the fuel and cannot be controlled by design.
3) *Data Required to Calculate the Efficiency:* The data required to calculate the efficiency of a boiler are: 1) Fuel analysis (H_2_, O_2_, S, C, humidity content, ash content), 2) Percentage oxygen or CO_2_ in flue gas F° gas temperature at °C (T_f_), 3) Ambient temperature in °C (T_a_), 4) Air humidity in kg/kg of dry air, 5) GCV fuel in kcal/kg, and 6) GCV ash in kcal/kg (solid fat) [31,34,41]. The efficiency is found by extracting heat loss fractions from 100 as shown in Fig. 12. Standards do not include blow-down loss in the process of determining efficiency. A detailed procedure for calculating the boiler’s efficiency is given below [[32], [33], [34], [35], [36], [37], [38], [39], [40], [41], [42]].

(5) $$=\frac{m\;x\;C_{p}\;x\;\left(T_{f}-T_{a}\right)}{GCV\;of\;fuel}\times 100$$

where, *m* is the mass of dry flue gas in kg/kg of fuel and *C*_*p*_ is the specific heat of flue gas (0.23 kcal/kg °C, m = Combustion products from fuel: CO_2_ + SO_2_ + Nitrogen in fuel + Nitrogen in the actual mass of air supplied + O_2_ in flue gas. (H_2_O/Water vapor in the flue gas should not be considered).

5) *Percentage of Heat Loss due to Water Evaporation:* The percentage of heat loss due to evaporation of water formed due to H_2_ in fuel can be expressed in Eq. (6) as:

(6) $$=\frac{9\;x\;H_{2}\;x\;{\{584+C}_{p}\;x\;\left(T_{f}-T_{a}\right)\}}{GCV\;of\;fuel}\times 100$$

where *H*_*2*_ is the kg of hydrogen in 1 kg of fuel, *Cp* is the specific heat of superheated steam (0.45 kcal/kg °C).

6) *Percentage of Heat Loss Due to Moisture Evaporation:* The percentage of heat loss due to evaporation of moisture present in fuel can be expressed in Eq. (7) as:

(7) $$=\frac{M\;x\;{\{584+C}_{p}\;x\;\left(T_{f}-T_{a}\right)\}}{GCV\;of\;fuel}\times 100$$

where *M* is the kg of moisture in 1 kg of fuel, *C*_*p*_ is the specific heat of superheated steam (0.45 kcal/kg°C), 584 is the latent heat corresponding to the partial pressure of water vapor.

7) *Percentage of Heat Loss Due to Moisture Present in Air:* The percentage of heat loss due to moisture present in air can be expressed in Eq. (8) as:

(8) $$=\frac{AAS\;x\;humidity\;factor\times C_{p}\times \left(T_{f}-T_{a}\right)}{GCV\;of\;fuel}\times 100$$

8) *Percentage of Heat Loss Due to Unburnt Fly Ash:* The percentage of heat loss due to unburnt fly ash can be represented in Eq. (9) as:

(9) $$=\frac{Total\;ash\;collected/K_{g}\;of\;fuel\;burnet\;x\;GCV\;of\;fly\;ash}{GCV\;of\;fuel}\times 100$$

9) *Percentage of Heat Loss Due to Unburnt Bottom Ash:* The percentage of heat loss due to unburnt bottom ash is expressed in Eq.(10) as:

(10) $$=\frac{Total\;ash\;collected\;K_{g}\;of\;fuel\;burnet\times GCV\;of\;bottom\;ash}{GCV\;of\;fuel}\times 100$$

10) *Percentage of Heat Loss Due to Radiation and Other Unaccounted Loss:* The percentage of heat loss due to radiation and other unaccounted loss is impacted by radiation loss and convection loss. Real radiation loss and convection loss are difficult to detect due to certain misalignments of the various surfaces, their inclination, airflow pattern, etc. For very small boilers, with a power of 10 MW, radiation and unbalanced losses may be between 1% and 2% of the total caloric value of fuel while for power of 500 MW values between 0.2% and 1% are standard. Loss may be properly considered depending on the circumstances. Thus the efficiency of the boiler can be calculated in Eq. (11) as:

(11) EfficiencyofBoiler(η)=100–(Thesevenaforementionedpercentageerrors)

11) *Boiler Evaporation Ratio (BER):* The Boiler Evaporation Ratio (BER) means kilogram of steam generated per kilogram of fuel consumed. In this case study, 1 kg of diesel can generate 15.2 kg of steam such that and using Eq. (12), the BER becomes 15.2. The BER can be expressed in Eq. (12) as:

(12) EvaporationRatio=HeatUtilizedforSteamGeneration/HeadAdditiontotheSteam

12) *The Furnace/Kiln Efficiency Analysis:* The efficiency is calculated using the gas analyzer.
13) *Calculation of Theoretical Air Requirement:* Theoretical air requirement can be expressed in Eq. (13) as:

(13) $$\left[\left(11.6\times C\right)+\left\{34.8\times \left(H_{2}-\frac{O_{2}}{8}\right)\right\}+\left(4.35\times S\right)\right]/100\;K_{g}/K_{g}\;of\;fuel$$

14) *Excess Air Supplied (EAS):* The EAS can be described in Eq. (14) as:

(14) $$EAS=\frac{O_{2}\%}{21-O_{2}}\times 100$$

15) *Calculation of Air Mass:* The air mass can be calculated in Eq. (15) as:

(15) $$Actual\;mass\;of\;air\;supplied/\;kg\;of\;fuel\;\left(AMAS\right)=\left(1+\frac{EAS}{100}\right)\times theoretical\;air$$

16) *Calculation of Heat Loss:* To evaluate the saving from insulated pipes the heat losses are calculated for uninsulated pipes, using the simplified formula in Eq. (16) for heat loss calculation.

(16) $$S=\left[10+\left(T_{s}-T_{a}\right)/20\right]\times \left(T_{s}-T_{a}\right)$$

where *S*: surface heat loss in kcal/h.m^2^ $,T_{s}$: hot surface temperature in °C $,T_{a}$: ambient temperature in °C.

17) *Calculation of Total Heat Loss:* Total heat loss of fuel is given in the following Eq. (17):

(17) THLF=S×A

where *A*: surface area in m^2^.

18) *Calculation of Equivalent Heat Loss:* The equivalentheatlossoffuelisgivenbyequation (18).

(18) EHLF(kg/year)=(THLF×yearlyhours)/(GCV×boilerefficiency)

### 2.5 Energy performance analysis

The basic measure of the facility’s energy performance is called the Energy Utilization Index (EUI) and it can be calculated by Eq. (19) by measuring the number of mega-joules (MJ) of energy used annually per square meter of facility space [37].

All energy forms had been identified inside the facility, the forms of energy used are electrical energy in KWh and diesel fuel in liters, number of MJ can be calculated. Each KWh contains 3.6 MJ, each 1 L of diesel fuel contains 39 MJ and the plant constructed area is 2390 m^2^.

(19) $$EUI=\frac{Total\;number\;of\;MJ\;of\;energy\;used\;annually}{constructed\;area\;m2}$$

Note the EUI is concerned with number of MJ. And it was not concerned with source and cost of each type of energy. The Energy Cost Index (ECI) Eq. (20) is used as a simpler and meaningful of energy efficiency. The Energy Cost Index adds up all dollar costs of energy used annually, and divides result by total square meters of conditioned space. Each 1 KWh of electricity cost 0.058008 JD, each 1 L of diesel fuel cost 0.6 JD, each 1 $ = 0.7 JD.

(20) $$ECI=\frac{Total\;cost\;in\;dollar\;\left(\$\right)\;for\;energy\;used\;annually}{constructed\;area\;m2}$$

ECI is very important indicator because it will reflect the saving directly especially if the factory decides to use another source of energy like PV system or concentrating solar power (CSP) system. In this case the EUI will not reflect these changes because it concerns with the number of MJ only.

## 3 Results and discussion

### 3.1 Information on key EA findings

**Fig. 13:**
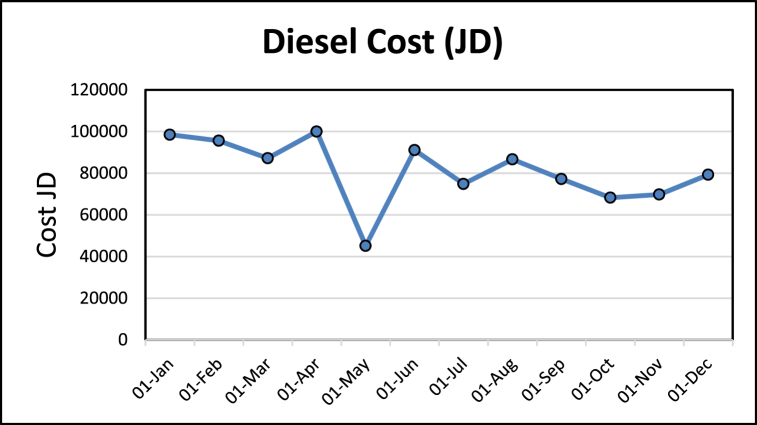
Diesel consumption cost.

**Fig. 14:**
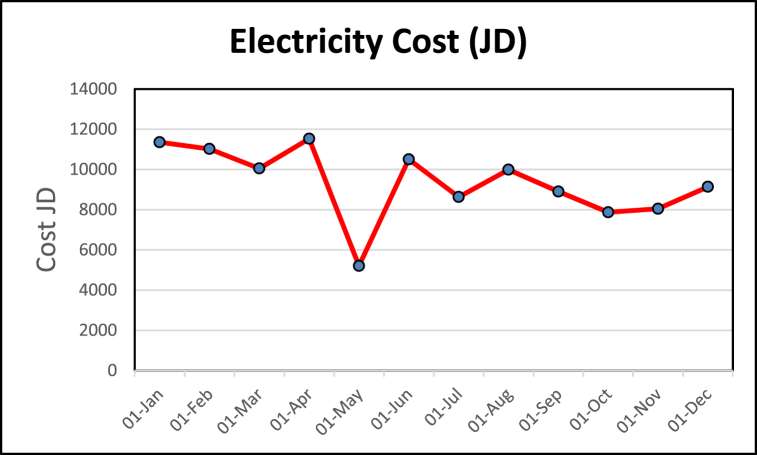
Electricity consumption cost.

EA is made to see the potential savings that could be done and if a more detailed audit is necessary. The energy consumption and production data are collected. In addition to that, the boiler efficiency was tested. It was revealed that diesel consumption is ten times more than electricity consumption, as shown in Fig. 13, Fig. 14 and concluded from Table 2 respectively. This depicts that savings in the boiler and steam distribution systems would be a major saving for the factory. The value of the standard deviation along with the coefficient of variation (CV%) are calculated in Table 2, it is clearly shown that the standard deviation is high for both the electrical and the diesel fuel bills due to the fluctuations in the market price, the diesel fuel varies on a monthly basis in Jordan, and in regard to the electrical bills the tariff varies in accordance with based on consumption amount and time in the day.

**Table 2:** Energy use and energy cost analysis.

Month	Energy Consumed (kWh)	JD/kWh	Electricity Cost (JD)	Diesel (liters)	Total Diesel Cost (JD)	Litre (JD)
18-Apr	198,733	0.058007	11,528	166666.67	100,000	0.6
18-May	89,729	0.058008	5205	75251.545	45150.927	0.6
18-Jun	181,117	0.058007	10,506	151891.02	91134.609	0.6
18-Jul	148,817	0.058004	8632	124797.57	74878.541	0.6
18-Aug	172,172	0.058006	9987	144387.55	86632.528	0.6
18-Sep	153,493	0.058009	8904	128730.02	77238.013	0.6
18-Oct	135,689	0.058008	7871	113795.37	68277.223	0.6
18-Nov	138,699	0.058011	8046	116325.44	69795.266	0.6
18-Dec	157,559	0.058004	9139	132127.55	79276.527	0.6
19-Jan	195,762	0.058004	11,355	164165.48	98499.285	0.6
19-Feb	189,986	0.058004	11,020	159322.2	95593.318	0.6
19-Mar	173,263	0.058004	10,050	145298.37	87179.024	0.6
Average			9353.583333		81137.93842	
Standard Deviation			1799.302969		15608.10952	
The coefficient of variation (CV%)			19.23650974		19.23651232	

#### 3.1.1 Key performance indicators

It can be further concluded that the use of diesel makes 90% of the energy consumption at the plant. The high demand for products explains the sudden rise in energy consumption in April, just the month before Ramadan. This makes the demand at this stage to be the highest. The decrease in energy consumption in August is explained by the fact that during Ramadan the production is the lowest. It has also been observed that the rise in diesel consumption in June is due to steam leaks and a major leak respectively. The leaks occur at the machine inlet, whereas the significant leaks occur at the inlet of the feedback tank.

1) Calculation of EUI: Referring to the data displayed in Table 2, the EUI can be found through (19) as 29394.84 (MJ/m^2^/yr.). The huge value of EUI came from diesel consumption. All the quantity of diesel was consumed by completely burning inside the boiler to generate the steam that is used in the production processes of food (Tahini and Halava). The ECI through (20) is 649.0725 USD/m^2^/yr.

### 3.2 Implementing the EA measures

Since the plant is a food production factory where electric motors and steam systems are used to produce food, the main energy consumption systems in the plant are electrically associated with motors, lighting, and small use of air-conditioning at the offices. Moreover, diesel is associated with the steam system utilized for the food production process and the use of steam pipes for heating the plant.

#### 3.2.1 Implementing the EA measures for the electrical consuming loads

Table 1, shows that all motors over 1 hp and with a utilization time of 2000 h per year or greater are likely candidates for replacement by high-efficiency motors. The motor that is forced into the rewinding process should be changed. The efficiency of the motor has been reduced by 2% after the rewound process. New motor models have higher efficiency than the old model with an estimated efficiency of 93–95%. This shows that there is a high potential saving of electrical energy by changing the old model of the motor with a new high-efficiency model [38,43,44]. According to the data above, motors # 4, 8, 12, 13, 14, 20, 22, 24, 25, and 28 should be changed. This can also be seen in Table 3 which shows the list of motors that should be changed with annual saving and simple payback period (SPP) calculations.

**Table 3:** List of motors that should be changed with energy saving calculation.

motor #	size kW	Working hours/year	Efficiency after rewinding process% (E_R_)	New efficiency model% (E_N_)	Price (Initial Cost) (JD)	annual saving (JD)	SPP (yr)	SPP (month)
4	4.8	2496	86	93	245	326.1509905	0.7512	9.01423
8	4	8640	88	93	180	547.2844575	0.3289	3.94676
12	4	8640	88	93	180	547.2844575	0.3289	3.94676
13	4	8640	88	93	180	547.2844575	0.3289	3.94676
14	4	8640	88	93	180	547.2844575	0.3289	3.94676
20	23	8640	84	93	2000	34121.23134	0.0586	0.70337
22	4	8640	88	93	180	547.2844575	0.3289	3.94676
24	6	8640	87	93	450	1494.652725	0.3011	3.61288
25	6	8640	87	93	450	1494.652725	0.3011	3.61288
28	6	8640	87	93	450	1494.652725	0.3011	3.61288

Table 3 shows the total SPP of the proposed motor replacement. It is evaluated to be on average 0.33576 (yr) with a financial saving of about 4166.776 JD/month.

**Fig. 15:**
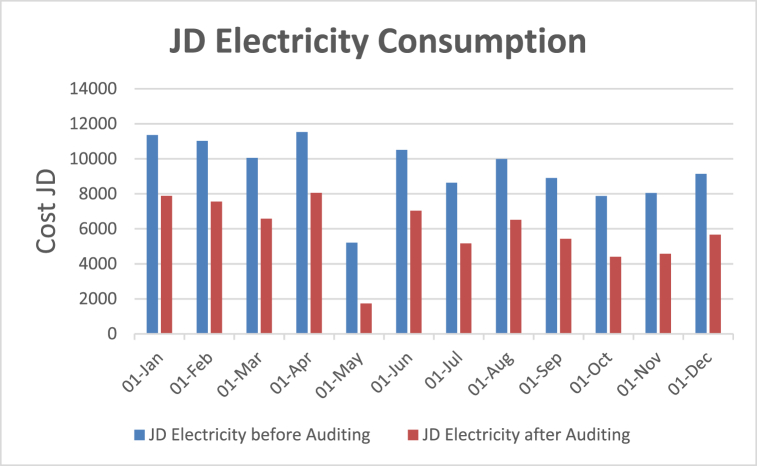
Bar graph-based energy cost analysis – Before and after auditing.

**Fig. 16:**
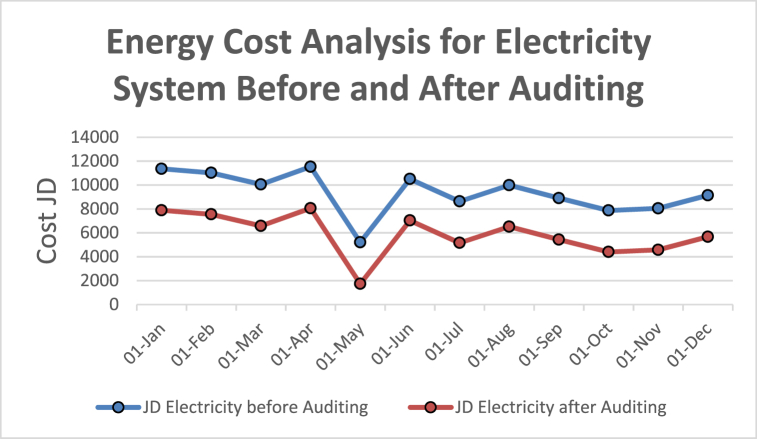
Plot based energy cost analysis.

Table 4 shows energy use and energy cost analysis for the electrical system after auditing respectfully. The energy cost analysis for the electricity consumption before and after the audit is shown in the graphs plotted in Fig. 15, Fig. 16 respectively. Table 4 reveals the tangible saving/month after motors replacement to be around 3472.314 JD/month. In other words, the reduction percentage in electrical power consumption which took place for the electrical system as indicated by the difference between the coefficient of variation (CV%) after the EA and the coefficient of variation (CV%) before the EA which is 11.357% due to the reduction of the average annual electrical power consumption which resulted from the energy audit.

**Table 4:** Energy use and energy cost analysis for electrical system after auditing.

Month	kWh	JD/kWh	JD Electricity before Auditing	JD Saving/Month	JD Electricity after Auditing
18-Apr	198,733	0.058007	11,528	3472.314	8055.686
18-May	89,729	0.058008	5205	3472.314	1732.686
18-Jun	181,117	0.058007	10,506	3472.314	7033.686
18-Jul	148,817	0.058004	8632	3472.314	5159.686
18-Aug	172,172	0.058006	9987	3472.314	6514.686
18-Sep	153,493	0.058009	8904	3472.314	5431.686
18-Oct	135,689	0.058008	7871	3472.314	4398.686
18-Nov	138,699	0.058011	8046	3472.314	4573.686
18-Dec	157,559	0.058004	9139	3472.314	5666.686
19-Jan	195,762	0.058004	11,355	3472.314	7882.686
19-Feb	189,986	0.058004	11,020	3472.314	7547.686
19-Mar	173,263	0.058004	10,050	3472.314	6577.686
Average					5881.269333
Standard Deviation (STD)					1799.302969
The coefficient of variation (CV%) of the electrical bill after EA%					11.357

Moreover, it is also recommended to reduce the monthly electricity bill to an absolute minimum. This is feasible by installing a PV system to feed the factory with the needed power and replacing all the lighting units and fixtures with light-emitting diode (LED) lighting units. Moreover, the two air conditioning units found in the two offices needs to be changed with energy-saving units containing inverters. The impact of the lighting and air-conditioning after replacement on the overall electrical bill is low so they are not included in the analysis.

#### 3.3.2 Implementing the EA measures for the boiler system

The following is the data for a diesel-fired steam boiler in the factory. Type of Boiler: Diesel Fired. Ultimate analysis of Oil: C = 84.0%, H_2_ = 12.0%, S = 3.0%, O_2_ = 1.0%, GCV of Oil = 10,600 kcal/kg, Steam Generation Pressure = 8 bar (g)-saturated, Enthalpy of steam = 664 k cal/kg, Feed water temperature = 70 °C. Percentage of Oxygen in flue gas = 7.1%, Percentage of CO_2_ in flue gas = 9.33%, Flue gas temperature (T_f_) = 185.2 °C. Ambient temperature (T_a_) = 17.2 °C. The humidity of air = 0.018 kg/kg of dry air. Diesel consumption rate = 162.8974 kg/h or 191.644 l/h, Density of diesel = 850 kg/m^3^. By applying (5)–(10), the boiler efficiency is 83.38%.

1) *Boiler Evaporation Ratio (BER):* In this case study, 1 kg of diesel can generate 15.2 kg of steam. The calculated BER by using (12) is 15.2.
2) *The Furnace Efficiency:* The following data is tabulated using the gas analyzer of type NOVA plus. The furnace efficiency is 90.6% as shown in Table 5.

**Table 5:** Gas analyzer readings.

O_2_ [%]	7.1
CO_2_ [%]	9.33
CO [ppm]	0
NO [ppm]	56
NO_2_ [ppm]	13
NO_x_ [ppm]	70
SO_2_ [ppm]	369
CH_4_ [%]	0.000
T_gas_ [C^°^]	185.2
T_air_ [C^°^]	17.2
Losses NCV [%]	9.4
Eff. NCV [%]	90.6
Eff. GCV [%]	--,-
Dewpoint [C^°^]	43.7
Air ratio	1.51
Exc.Air [%]	51
Draft [hpa]	−0.06
CO [ppm/ref0%O_2_]	0

#### 3.2.3 *M*easures to improve the boiler performance are implemented in accordance with related references and research as follows bearing in mind that maintenance workers do the measures and would not cost extra money

1) Providing an Accurate Amount of Combustible Air: Increasing the combustion efficiency ensure that the fuel is completely burned by providing the right amount of combustible air given in (13)–(15). Too much air will reduce efficiency due to the air traps. There is a recommended amount of excess air in each type of fuel combustion, and it is 15% of the oil system. Boiler efficiency can increase by 1% for every 15% reduction in excess air. A study of the gas analyzer showed that the excess air is 51%, by reducing the amount of excess air by 15% boiler efficiency will increase by 2% [[39], [40], [41], [42], [43], [44], [45]]. After adjusting the amount of air, the boiler efficiency will increase, and thus the fuel consumption will decrease by the same percentage. The amount of fuel saving percentage is equal to (51%–15%)/15% = 2.4%. Also, ($m{}^{\circ}{{}^{\circ}}_{diesel}$) _after_ = 162.8974 × (100–2.4) % = 159.0815 kg/h = 187.15 l/h. The amount of diesel fuel saved is the difference before and after applying energy auditing which is 4.489 l/h. And thus, the amount of saving JD/month = 4.489 × 24 x 30 × 0.6 JD = 1939.248 JD/month because in Jordan the diesel litre price is 0.61 JD.
2) Fixing all the Steam Leaks: Fixing all the steam leaks, hence some inlets from the pipes to the machines are leaking, it is visible and can be detected easily. A major leak is from the steam line that is entering the feed tank. Fixing the steam leaks is not expensive and can be done by the maintenance workers at the plant by just changing the leaking pipes or fittings. There are seven points of the steam leak that are detected by visual inspection, the amount of fuel-saving from repair and fixing all the points of the leak is estimated to be 3–5% [[39], [40], [41], [42], [43], [44], [45]]. Diesel fuel consumption before maintaining all steam leak points would be reduced to 3% ($m{}^{\circ}{{}^{\circ}}_{diesel}$) after = (191.644 l/h) × 0.97 = 185.894 l/h, so the amount of diesel fuel saved 5.749 l/h and as a result the amount of saving JD/mo. = 5.749 × 24 x 30 × 0.6 JD = 2483 JD/mo.
3) Reduced Heat Losses from Uninsulated Pipes: Reduced heat losses from uninsulated pipes as displayed in Table 6 will save a huge amount of fuel consumption. The type of insulation used inside the factory was 65 mm glass wool with aluminum cladding. There are some uninsulated pipes in which the insulation was broken or removed. The length and diameter of uninsulated pipes were measured. To calculate the losses, the heat losses after insulation are calculated as follows: Repeat the same procedure by changing the surface temperature to 65 °C (Surface temperature after insulation) so THLF after insulation = 2699.03 kg/yr and ΔTHLFF = 25578.03 kg/yr which is equivalent to 30091.8 l/yr which yields Fuel cost = Δ THLF × 0.6JD = 18055.08 JD/yr. = 1504.59 JD/mo.

**Table 6:** Uninstalled pipe- Reduced heat losses.

Uninsulated pipe $T_{s}$ = 170 °C & $T_{a}$ = 25 °C					
Pipe Diameter (mm)	Length(m)	space area A (m^2^)	S (kcal/h.m^2^)	Hs (kcal/h)	HF(kg/yr.)
25.4 (1 inch)	65	5.184	2501.25	12966.83	**28277.06**
31.75 (1.25 inch)	40	3.987	2501.25	9974.48	
50.8 (2 inch)	15	2.392	2501.25	5984.69	
				**Total = 28,926**
4) *Fixing the Steam Traps:* Fixing the steam traps that is stuck sometimes is done locally by the maintenance workers and would not cost extra money. Fuel-saving from maintaining and checking the steam traps is estimated to be 10% and if the factory decides to install the automated monitor on steam traps, then this will generate extra saving. This extra saving is estimated to be 5% [37,39,[45], [46], [47], [48]]. Diesel fuel consumption before maintaining steam traps is ($m{}^{\circ}{{}^{\circ}}_{diesel}$) _before_ = 191.644 l/h, whereas diesel fuel consumption after maintaining steam traps would be reduced to 10% [32,39]. So ($m{}^{\circ}{{}^{\circ}}_{diesel}$) after = (191.644 l/h) × 0.9 = 172.479 l/h. This yields the amount of diesel fuel saved = 191.644–172.479 = 19.164 l/h. This gives the amount of saving JD/mo. = 19.164 × 24 × 30 × 0.6 JD = 8279.02 JD/mo. The improvements discussed above which took place on the diesel boiler system are depicted in Table 7 associated with Fig. 17, Fig. 18.

**Table 7:** Energy use and energy cost analysis for boiler system after auditing.

Month	JD Diesel before Auditing	Litre (JD)	JD Saving/mo.	JD Diesel after Auditing	Percentage Saving (%)
18-Apr	86632.52837	0.6	14205.85	72426.67837	16.3978
18-May	91134.6093	0.6	14205.85	76928.7593	15.5878
18-Jun	74878.54059	0.6	14205.85	60672.69059	18.9719
18-Jul	100,000	0.6	14205.85	85794.15	14.2059
18-Aug	45150.92722	0.6	14205.85	30945.07722	31.463
18-Sep	77238.01268	0.6	14205.85	63032.16268	18.3923
18-Oct	68277.22347	0.6	14205.85	54071.37347	20.8061
18-Nov	69795.26617	0.6	14205.85	55589.41617	20.3536
18-Dec	79276.52716	0.6	14205.85	65070.67716	17.9194
19-Jan	98499.28503	0.6	14205.85	84293.43503	14.4223
19-Feb	95593.31757	0.6	14205.85	81387.46757	14.8607
19-Mar	87179.02374	0.6	14205.85	72973.17374	16.295
18-Apr	102,620	0.6	14205.85	88414.15	13.8432
Average					68584.55472
Standard Deviation (STD)					16087.58441
The coefficient of variation (CV%) of the diesel fuel bill after EA%					4.024

**Fig. 17:**
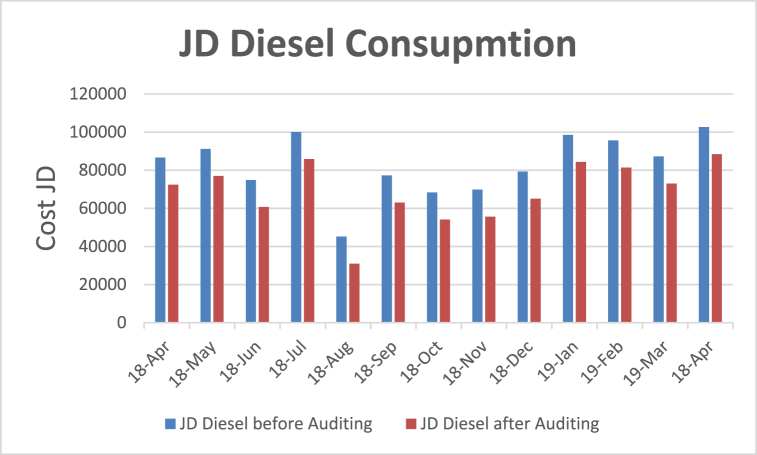
Energy cost analysis for boiler system before and after auditing - Energy saving per month.

**Fig. 18:**
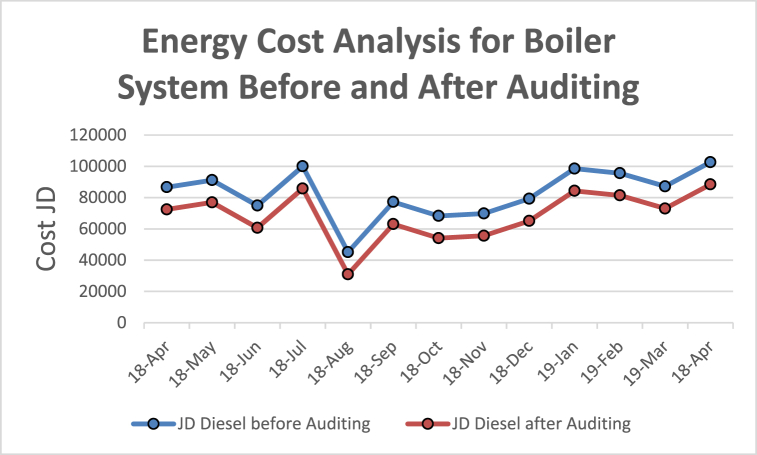
Energy cost analysis for boiler system before and after auditing – Energy saving per month.

It has been verified that in this definite case, the enhancement of the factory energy usages covers and the optimization of the performances of the current boiler systems can limit a decrease of diesel consumption up to 16.63% on average per month with an anticipated monthly financial saving of the order of 14205.85 JD/month. In other words, the reduction percentage in diesel fuel consumption which took place for the boiler system as indicated by the difference between the coefficient of variation (CV%) after the EA and the coefficient of variation (CV%) before the EA which is 4.024% due to the reduction of the average annual diesel fuel consumption which resulted from the energy audit.

### 3.3 Environmental impact mitigation

The amount of kg CO_2_ saving/yr. that results from applying energy audit measures for the diesel boiler system is as follows [33]. Each kg of diesel fuel burning gives 3.2 kg CO_2_. Amount of diesel fuel saving per month = 23677.09 l/mo. Amount of diesel fuel saving per year = 284125.08 l/yr = 241506.31 kg/yr. CO_2_ saving per year = 241506.31 (kg/yr.) × 3.2 = 772820.21 kg/yr = 772.82021 Ton CO_2_/yr.

#### 3.3.1 Further discussions

The further discussions in this part show that the present data were collected from factory-registered data at the time of this energy audit research to acquire 1) accurate evaluations, 2) measurements, 3) analyses, and 4) applications for energy accounting and effective factory energy management. The information gathered is primarily used to pinpoint energy-saving measures (ECMs) that are impairing plant performance as well as to quantify and confirm energy savings.

#### 3.3.2 Potential savings

The potential savings can be attained by adhering to the suggestions of an expert in the energy audit report by implementing those actions and keeping up with new technologies in the energy audit domains. It is suggested to 1) gather data from utility bills, 2) examine meter data, 3) search for cost-saving alternatives, and 4) track your progress to maintain ongoing energy savings.

#### 3.3.3 Analysis of energy use and energy savings

A case study of a condiment industry in India [29] serves as similar research in the same field that validates the results of the energy audit and the actions performed in this research. As part of an energy audit technique utilized by many businesses, a qualified team keeps an eye on, investigates, and analyses the energy flow inside the facility. This is how the energy audit was put into practice. To choose the optimal line of action for energy saving, this is done. It entails a variety of steps, including lowering carbon footprint, energy costs, and usage. Energy auditing techniques are used at the plant to identify ways to reduce facility operating expenses and/or the amount of energy used to produce one unit of output. The three phases of the audit are 1) the pre-audit phase, 2) the audit phase, and 3) the post-audit phase. The following topics are included in an energy audit coverage: 1) Boilers, 2) lighting, and 3) other energy-producing or energy-converting machinery. The main goal of an energy audit is to find ways to lower operating expenses or energy usage per unit of output. The next area for further study at the factory for the energy audit is suggested to be energy distribution networks, water, condensate, compressed air, and other energy generation/conversion equipment, such as furnaces, pumps, fans, compressors, and transformers.

## 4 Conclusion

An efficient energy cost reduction is vital for expanding the effectiveness of an enterprise. This can be achieved by the controlled means of an EA. The awareness of EA is not only a tangible prospect for the enterprises but also one of the prioritized requirements anticipated by the industrial giants. In this paper, an effective EA of an industrial site for food production is implemented. It has been ensured here how the energy audit allows for gathering information that is very valuable to describe a factory’s energy state. It further utilizes the energy steadiness of the factory for investigation. By means of the factory energy state, it is possible to 1) revise the influence of probable enhancements of the site, 2) attain and mitigate environmental pollution, and 3) shrink energy budgets. A sequence of potential energy-saving activities has been recommended. For each recommendation, the expected energy saving per month has been calculated by using the factory energy-efficient measures. The pay-back time linked to a motor has also been calculated. The study has also shown that it is possible to implement a series of energy tradeable measures, such as 1) thermal insulation, 2) leaking treatment, and 3) steam traps of boilers. This can yield a saving of about 14205.85 JD/month. The factory will use the outcomes of this energy audit for the characterization of its future energy-saving policy. It can also be structured for implementation on other factory sites too.

## Author contribution statement

Derar Al Momani, Tha’er O. Sweidan, Yousef Al Turk, Mohammed I. Abuashour, Haris M. Khalid: Conceived and designed the experiments; Performed the experiments; Analyzed and interpreted the data; Contributed reagents; Materials; Analysis tools or Data; Wrote the Paper.

S. M. Muyeen, Zafar Said, M. Hasanuzzaman: Analyzed and interpreted the data; Contributed reagents; Materials; Analysis tools or Data; Wrote the Paper.

## Funding statement

S M Muyeen was supported by Qatar National Library.

## Data availability statement

Data included in article/supplementary material/referenced in article.

## Declaration of interest’s statement

The authors declare no conflict of interest.

## Nomenclature

**Acronyms**
AMAS: Actual Mass of Air Supplied
ASME: American Society for Mechanical Engineers
BER: Boiler Evaporation Ratio
BS: British Standard
CSP: Concentrating Solar Power
CP: Control Point
CCP: Critical Control Point
ER: Efficiency after Rewinding Process Percentage
EA: Energy Audit
ECI: Energy Cost Index
ECM: Energy Saving Measures
EUI: Energy Utilization Index
EAS: Excess Air Supplied
GCV: Gross Caloric Value
JD: Jordanian Dinar
LED: Light Emitting Diode
LF: Load Factor
MJ: Mega Joules
EN: New Efficiency Model Percentage
PTC: Power Test Code
REI: Renewable Energy Integration
SPP: Simple Payback Period
THLF: Total Heat Loss of Fuel
VFD: Variable Frequency Drive
V2G: Vehicle-to-Grid

**Symbols**
*T*_*a*_: Ambient Temperature
*T*_*f*_: Flue Gas Temperature
*T*_*s*_: Hot Surface Temperature
*A*: Surface Area
*C*_*p*_: Specific Heat of Flue Gas
