# Corrigendum to “Energy saving potential analysis applying factory scale energy audit – A case study of food production” [Heliyon 9 (3), (March 2023) Article e14216]

**DOI:** 10.1016/j.heliyon.2023.e16551

**Published:** 2023-06-12

**Authors:** Derar Al Momani, Yousef Al Turk, Mohammed I. Abuashour, Haris M. Khalid, S.M. Muyeen, Tha'er O. Sweidan, Zafar Said, M. Hasanuzzaman

**Affiliations:** a Department of Allied Engineering Sciences, Faculty of Engineering, The Hashemite University, Zarqa, Jordan; b Alternative Energy Technology Department, Al-Zaytoonah University of Jordan, Queen Alia Airport St 594, Amman, Jordan; c Renewable Energy Center, Electrical Engineering Department, Faculty of Engineering, The Hashemite University, Zarqa, Jordan; d Department of Electrical and Electronics Engineering, Higher Colleges of Technology, Sharjah, 7947, United Arab Emirates; e Department of Electrical and Electronic Engineering Science, University of Johannesburg, Aukland Park 2006, South Africa; f Department of Electrical Engineering, University of Santiago, Avenida Libertador 3363, Santiago, RM, Chile; g Department of Electrical Engineering, Qatar University, Doha, 2713, Qatar; h Sustainable and Renewable Energy Engineering Department, College of Engineering, University of Sharjah, PO Box 27272, Sharjah, United Arab Emirates; i U.S.-Pakistan Center for Advanced Studies in Energy (USPCAS-E), National University of Sciences and Technology (NUST), Islamabad, Pakistan; j Department of Industrial and Mechanical Engineering, Lebanese American University (LAU), Byblos, Lebanon; k Higher Institution Center of Excellence (HICoE), UM Power Energy Dedicated Advanced Center (UMPEDAC), University of Malaya, Jalan Pantai Baharu, 59990, Kuala Lumpur, Malaysia; l College of Engineering and Information Technology, University of Dubai, Academic City 14143, Dubai, United Arab Emirates

In the original published version of this article, the authors have requested to change the institutional information. The institutional information of the authors have been updated. *The correct version of the institutional information of the authors can be found below*:

Mohammed I. Abuashour institutional information has been changed from:

Renewable Energy Centre, The Hashemite University, Zarqa, 13115, Jordan

to:

Renewable Energy Centre, Electrical Engineering Department, Faculty of Engineering, The Hashemite University, Zarqa, Jordan.

And.

Haris M. Khalid L institutional information has been changed from:

Department of Electrical and Electronics Engineering, Higher Colleges of Technology, Sharjah 7947, United Arab Emirates

To.

College of Engineering and Information Technology, University of Dubai, Academic City 14143, Dubai, United Arab Emirates

The authors/publisher apologize for the errors. Both the HTML and PDF versions of the article have been updated to correct the errors.

## Declaration of competing interest

The authors declare that they have no known competing financial interests or personal relationships that could have appeared to influence the work reported in this paper.

